# miR-455 Inhibits the Viability and Invasion by Targeting RAB18 in Hepatocellular Carcinoma

**DOI:** 10.1155/2021/9923454

**Published:** 2021-05-27

**Authors:** Chenghong Wang, Guicai Zhu, Miaolin Yu, Xiufang Mi, Honghua Qu

**Affiliations:** ^1^Department of Clinical Laboratory, Yantaishan Hospital, Yantai 264000, China; ^2^Department of Surgery, Rizhao Hospital of TCM, Rizhao 276800, China; ^3^Department of Chinese Medicine, Qingdao Central Hospital, Qingdao University, Qingdao 266042, China; ^4^Department of Internal Medicine, Zhangqiu District People's Hospital, Jinan 250200, China; ^5^Department of Medical Insurance Office, Qilu Hospital of Shandong University, Jinan 250012, China

## Abstract

**Background:**

Hepatocellular carcinoma (HCC) has been regarded as the fifth most common cancer worldwide with a low prognosis. miR-455 usually played the role of a tumor suppressor in multiple cancers. The aim of this study was to investigate the roles of miR-455 in HCC.

**Materials and Methods:**

Cell viability and invasion were measured by CCK8 and Transwell assays. Luciferase reporter assay was performed to verify that miR-455 directly binds to the 3′-noncoding region (UTR) of RAB18 mRNA in Huh7 cells.

**Results:**

The expression of miR-455 was lower in HCC tissues and cell lines than in nontumor tissues and normal cell line, and downregulation of miR-455 was connected with worse outcome of HCC patients. miR-455 suppressed cell proliferation in vitro and in vivo, and it inhibited the abilities of cell invasion and EMT in HCC. RAB18 was upregulated in HCC tissues and cell lines, and the expression of RAB18 was regulated by miR-455. RAB18 reversed partial roles of miR-455 on cell viability and invasion in HCC.

**Conclusion:**

miR-455 inhibited cell viability and invasion by directly targeting the 3′-UTR of RAB18 mRNA of hepatocellular carcinoma.

## 1. Introduction

Hepatocellular carcinoma (HCC), the third major cause of cancer-related death, is the fifth most common cancer worldwide [1]. In recent years, the increase in the incidence of HCC has been the result of a combination of factors, especially the phenotype caused by hepatitis B or C virus (HBV or HCV) infection [2, 3]. Despite significant advances in treatment and diagnosis, surgery is the primary treatment for patients with HCC. About half of HCC cases are advanced unresectable HCC, resulting in a poor prognosis [4]. Thus, it is necessary to investigate the biomarkers for the treatment and the pathogenesis of HCC.

MicroRNAs (miRNAs) are small noncoding endogenous RNAs containing 19 to 25 nucleotides that promote posttranscriptional control in regulating the expression of target gene by binding to the 3′-UTR sequences of its mRNA [5]. Recently, increasing evidences elucidated that miRNAs was involved in the cancer pathogenesis, including cell proliferation, metastasis, and apoptosis [6]. Most reports found that multiple miRNAs that include miR-548a, miR-1246, miR-632, and miR-5692a played pivotal roles in HCC [7–10]. miR-455 has been reported to act as a tumor suppressor to inhibit cap-dependent translation and the proliferation in prostate cancer [11]. Also, in gastric cancer, miR-455 inhibited human cell proliferation and invasion and promoted cell apoptosis [12]. In addition, miR-455 inhibited cell viability, while it induced cell apoptosis in colorectal cancer [13]. However, miR-455 promoted cell invasion and migration in triple-negative breast cancer [14]. Thus, the pivotal roles of miR-455 in cell viability and metastasis in HCC still needed to be explored.

RAB18, a member of Ras-related small GTPases family, belongs to members of the Ras oncogene superfamily of small guanosine triphosphatases [15]. Accumulating evidences have elucidated connection between the expression of GTPases members and several diseases, including colorectal cancer, lung cancer, and prostate cancer [16–18]. RAB18 regulates membrane trafficking in organelles and transport vesicles, leading to a reduction in mature LDs and lipid storage [19]. RAB18 binds to NS5A to improve the interaction between sites of viral replication and lipid droplets [20]. RAB18 is reduced in pituitary tumors, resulting in acromegaly and restoration of excessive growth hormone hypersecretion [21]. RAB18 was associated with lipogenesis, lipolysis, and obesity in adipocytes [22]. However, the functions of RAB18 in HCC remain unclear; thus, in the present study, we discovered that miR-455 inhibited cell viability, invasion, and EMT by directly targeting to the 3′-UTR of RAB18 mRNA in hepatocellular carcinoma.

## 2. Materials and Methods

### 2.1. Patients and Tissue Samples

A cohort of 98 patients who underwent HCC were harvest from Yantaishan Hospital during the period from January 2016 to November 2018. None of the patients received preoperative treatment such as chemotherapy and radiotherapy before surgery. All the fresh tissues were snap-frozen in liquid nitrogen and stored at −80°C until RNA extraction. The project protocol was reviewed and approved by the Ethics Committee of Zhangjiagang Hospital. All participants signed the written informed consents before inclusion in this study.

### 2.2. Cell Culture

HCC cell lines HCC-LM3, Huh7, and Bel-7402 and a normal liver cell L-O2 were obtained from American Type Culture Collection (ATCC, Rockville, MD, USA). All cells were cultured with Dulbecco's modified Eagle's medium (DMEM, Invitrogen, CA, USA) supplemented with 10% FBS (Gibco, USA) at 37°C in a humidified atmosphere containing 5% CO_2_.

### 2.3. Western Blot

Radioimmunoprecipitation assay (RIPA, Thermo Fisher Scientific, Waltham, MA, USA) lysis buffer containing PMSF was employed to separate total protein on ice for 37°C. After centrifugation at 12,000 × g for 20 min at 4°C, the concentration of the protein was then quantified using BCA Protein Assay Reagent Kit (Beyotime, Shanghai, China). Equal amounts of protein for each sample were loaded and separated on a 10% sodium dodecyl sulphate-polyacrylamide gel (SDS-PAGE) before being transferred onto a polyvinylidene difluoride (PVDF, Roche Diagnostics) membrane. After blocking in 5% skim milk powder at room temperature for 1 h, the membrane was incubated by primary antibodies overnight at 4°C. The primary antibodies were RAB18 (1 : 1000, Santa Cruz Biotechnology, Santa Cruz, CA, USA), E-cadherin, N-cadherin, and glyceraldehyde-3-phosphate dehydrogenase (GAPDH). Next, horseradish peroxidase- (HRP-) conjugated secondary antibody (1 : 5000) was conducted to incubate the membrane at room temperature for 1 h. The Enhanced Chemiluminescence (ECL) Kit (KeyGen Biotech, China) was applied to measure the bands and imaged on a Tanon-5200 Chemiluminescent Imaging System (Millipore, Bedford, MA, USA).

### 2.4. RNA Isolation and qRT-PCR

TRIzol reagent (Invitrogen, USA) was used to extract the total RNAs from tissues and cell lines. The miRNA was reverse-transcribed using miRNA first-strand complementary DNA (cDNA) synthesis kit (Poly A Tailing; Sangon, China), while mRNA was reverse-transcribed using RevertAid First-Strand cDNA Synthesis Kit (Thermo Fisher Scientific). Subsequently, qRT-PCR was carried out using SYBR Premix Ex Taq kit (Takara) on an ABI PRISM 7900 Sequence Detection System (Applied Biosystems). The 2^−ΔΔCq^ method was applied to analyze the miRNA or mRNA expression with GAPDH or U6 was the normalization. The primer pairs are shown in Table 1.

### 2.5. Cell Proliferation Assay

The cell proliferation was calculated using cell counting kit-8 (CCK-8) assay (Dojindo Laboratories, Kumamoto, Japan) in 96-well plates. After 24, 48, 72, or 96 h of culture, CCK-8 solution was added to each well and cultivated for 3 h at 37°C. The plates were shook for 20 min, followed by measuring the absorbance at 450 nm using automatic multiwell spectrophotometer (Bio-Rad, Richmond, CA, USA).

### 2.6. Invasion Assay

Invasive ability was evaluated using Transwell insert (Corning Incorporated, Corning, New York) covered with Matrigel (BD Biosciences). Briefly, the cells were suspended in DMEM medium without FBS and the cell suspension was placed in the upper well, while the bottom well was filled with medium with 20% FBS that acted as chemoattractant. After incubation at 37°C with 5% CO_2_ for 24 h, the cells that failed to pass through the membrane were removed with cotton swabs. Meanwhile, the cells that passed through the membrane were fixed with 100% methanol for 15 min and stained using 0.1% crystal violet solution for 30 min. Finally, a microscope (Olympus, Tokyo, Japan) was utilized to count the invaded cells.

### 2.7. Cell Transfection

The miR-455 mimic, miR-455 inhibitor, and RAB18 overexpression plasmids, as well as corresponding negative control, were obtained from GenePharma (Shanghai, China). Cells were placed in a 6-well plate, and the transfections of special vectors were performed using Lipofectamine 2000 Reagent (Invitrogen, Carlsbad, CA, USA) following the manufacturer's instruction.

### 2.8. Dual-Luciferase Reporter Gene Assay

TargetScan predicted the miR-455 binding site at 3′-UTR of RAB18 mRNA. The putative sequences of miR-455 on RAB18 mRNA were mutated from GCACAUA to CGUGUAU. The wild type or the mutated sequences of RAB18 mRNA were cloned into pmiRGLO Vector, which were designated as pmiRGLO-RAB18-WT (WT) and pmiRGLO-RAB18-MUT (MUT), respectively. The Huh7 cells were seeded and cotransfected with pmiRGLO-RAB18-WT or pmiRGLO-RAB18-MUT plasmid and miR-455 mimic or miR-455 NC using Lipofectamine 2000 (Invitrogen, Carlsbad, CA, USA). Firefly luciferase activity was assessed by dual-luciferase reporter assay system (Promega, Madison, WI, USA), with Renilla luciferase activity acting as normalization.

### 2.9. Construction of the Mice Xenograft Model

Four-week-old nude mice were purchased from Vital River Laboratory Animal Technology (Beijing, China). Huh7 cells that transfected miR-455 mimic or control plasmids were subcutaneously inoculated in the mice to build xenograft tumor model. The volumes were calculated as 1/2 × length × width^2^ of the tumor, which were measured every 3 days. After cultivation for 26 days, the mice were executed and the tumors dissected out. The animal study was also approved by the Research Ethics Committee of Zhangjiagang Hospital.

### 2.10. Statistical Analysis

The data were indicated as means ± SD. The SPSS statistical software version 19.0 (IBM Corp, Armonk, NY, USA) and GraphPad Prism 7.0 (GraphPad, San Diego, CA) were used to analyze all the data. The differences between two or more groups were compared by Student's *t*-test or one-way analysis of variance (ANOVA) followed by the Student-Newman-Keuls post hoc test. Values of *P* < 0.05 were considered to be statistically significant.

## 3. Results

### 3.1. The Expression of miR-455 in HCC

**Figure 1:**
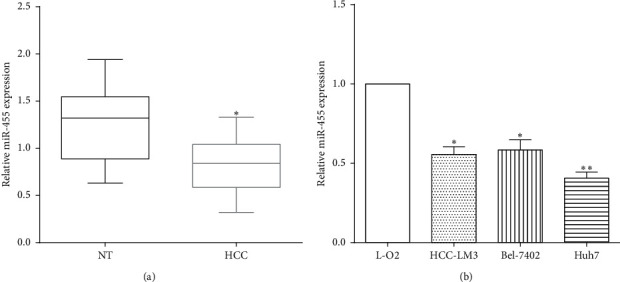
The expression of miR-455 in HCC. (a) The expression of miR-455 was low in HCC tissues versus noncancerous tissues vs NT, ^∗^*P* < 0.05. (b) The expression of miR-455 was lower in HCC cells than in normal cells vs L-O2, ^∗^*P* < 0.05; ^∗∗^*P* < 0.01.

To investigate the functions of miR-455 in HCC, qRT-PCR was conducted to measure the expression of miR-455 in tissues or cell lines. As a result, miR-455 had a low expression in HCC tissues versus noncancerous tissues (*P* < 0.05) (Figure 1(a)). The expression of miR-455 was also evaluated in HCC cell lines HCC-LM3, Huh7, and Bel-7402 and normal liver cell L-O2. As expected, the expression of miR-455 was lower in HCC cell lines HCC-LM3 (*P* < 0.05), Huh7 (*P* < 0.01), and Bel-7402 (*P* < 0.05) than in L-O2 cells (Figure 1(b)).

### 3.2. miR-455 Inhibits Cell Viability of HCC In Vitro and In Vivo

**Figure 2:**
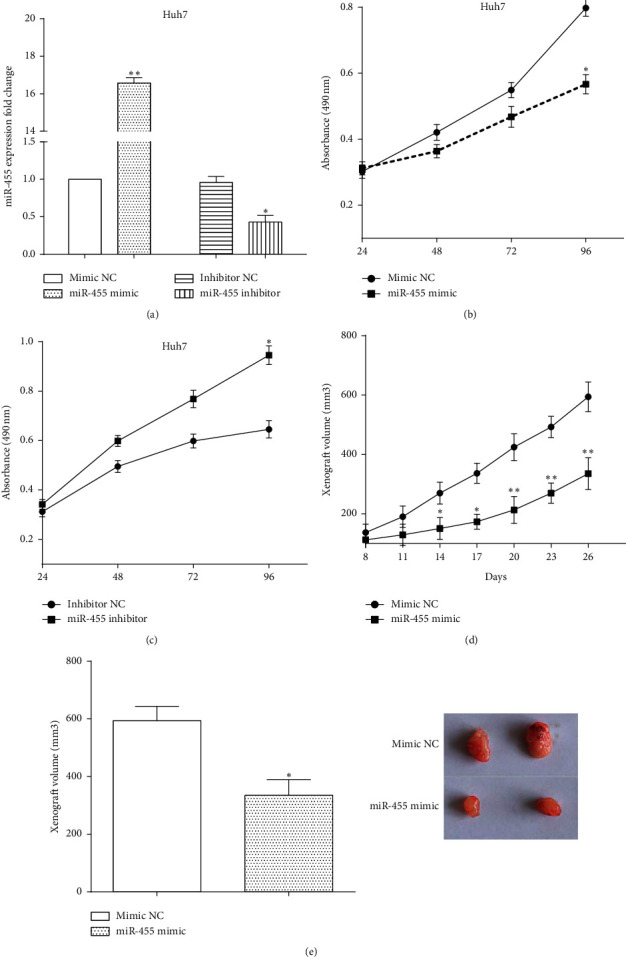
miR-455 inhibits cell viability of HCC in vitro and in vivo. (a) miR-455 mimic and miR-455 inhibitor were used to upregulate or downregulate the expression of miR-455 vs mimic NC or inhibitor NC, ^∗^*P* < 0.05, ^∗∗^*P* < 0.01. (b) The proliferative ability was decreased after transfection with miR-455 mimic vs mimic NC, ^∗^*P* < 0.05. (c) miR-455 inhibitor increased proliferative ability in comparison with normal control vs inhibitor NC, ^∗^*P* < 0.05. (d) miR-455 suppressed the growth of HCC in vivo vs inhibitor NC, ^∗^*P* < 0.05; ^∗∗^*P* < 0.01. (e) Tumors of miR-455 overexpressed group had a smaller volume than that of control group vs inhibitor NC, ^∗^*P* < 0.05.

To explore the roles of miR-455 in cell proliferation, miR-455 mimic and miR-455 inhibitor were utilized to upregulate (*P* < 0.01) or downregulate (*P* < 0.05) the expression of miR-455 in HCC cells Huh7, which was measured by RT-qPCR (Figure 2(a)). CCK8 assay showed that the proliferative ability was decreased by transfecting miR-455 mimic (*P* < 0.05) (Figure 2(b)). On the contrary, miR-455 inhibitor increased the proliferative ability in comparison with normal control (*P* < 0.05) (Figure 2(c)).

In addition, Huh7 cells stably transfected with miR-455 mimic had a slower growth rate in vivo (Figure 2(d)). After 26 days of cultivation, the nude mice were executed and the volume of the xenograft tumor was calculated. Not unfortunately, tumors of miR-455 overexpressed group had a smaller volume than that of control group (*P* < 0.05) (Figure 2(e)). All results elucidated that miR-455 suppressed the growth of HCC in vitro and in vivo.

### 3.3. miR-455 Impairs Cell Invasion and the EMT in HCC Cells

**Figure 3:**
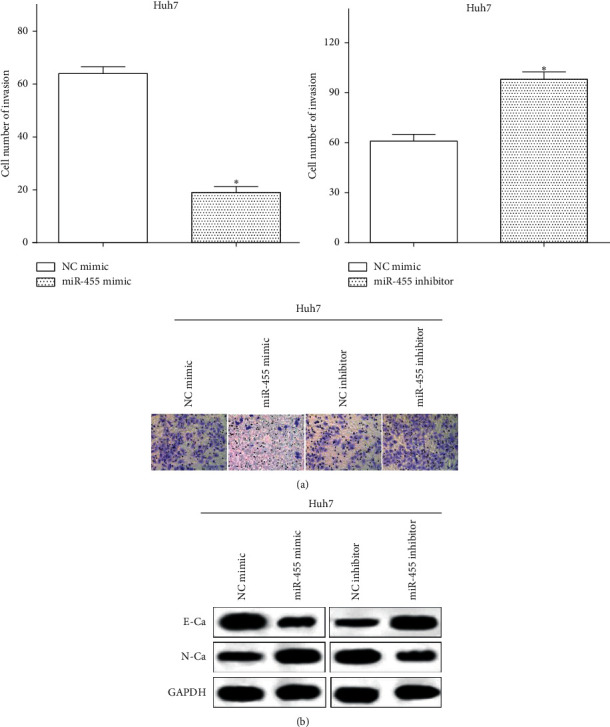
miR-455 impairs cell metastasis of HCC cells. (a) miR-455 mimic reduced the invasive ability, while miR-455 inhibitor improved that in Huh7 cells vs mimic NC, ^∗^*P* < 0.05. (b) miR-455 inhibited the EMT ability in Huh7 cells.

Transwell assay was performed to calculate cell invasion in Huh7 cells transfected with miR-455 mimic or miR-455 inhibitor. As expected, the invasive ability was reduced by miR-455 mimic (*P* < 0.05), while it was improved by miR-455 inhibitor in Huh7 cells (*P* < 0.05) (Figure 3(a)). Furthermore, proteins associated with EMT markers, such as E-cadherin and N-cadherin, were measured using western blot. As a result, the expression of N-cadherin was inhibited, while the expression of E-cadherin was improved by miR-455 mimic. On the contrary, miR-455 inhibitor enhanced the expression of N-cadherin, whereas it decreased the expression of E-cadherin in Huh7 cells (Figure 3(b)).

### 3.4. miR-455 Directly Targets the 3′-UTR of RAB18 mRNA

**Figure 4:**
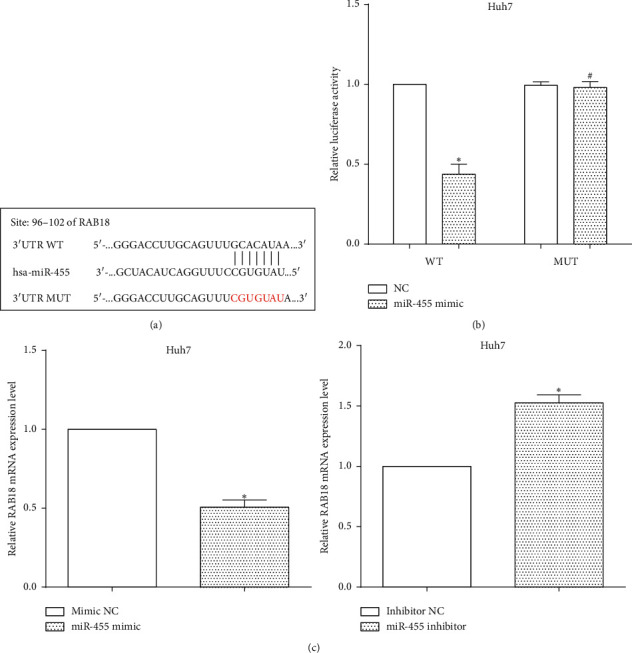
miR-455 targets RAB18 and regulates its expression. (a) TargetScan predicted that RAB18 was a potential target of miR-455. (b) miR-455 mimic reduced the luciferase ability of wild type mRNA 3′-UTR, whereas it did not alter the mutant 3′-UTR vs WT-NC, ^∗^*P* < 0.05 and vs MUT-NC, ^#^*P* < 0.05. (c) The expression of RAB18 was decreased by miR-455 mimic, while it was promoted by miR-455 inhibitor in Huh7 cells vs mimic NC, ^∗^*P* < 0.05 and vs inhibitor NC, ^∗^*P* < 0.05.

TargetScan predicted RAB18 was a potential target of miR-455 at 96–102 on the 3′-UTR. To verify miR-455 binding to the 3′-UTR of RAB18 mRNA, the putative binding sequences were mutated from GCACAUA to CGUGUAU, as shown in Figure 4(a). The miR-455 mimic and the wild type or the mutant 3′-UTR were cotransfected in Huh7 cells, followed by calculating the luciferase ability. As expected, miR-455 mimic reduced the luciferase ability of wild type mRNA 3′-UTR (*P* < 0.05), whereas it did not alter the mutant 3′-UTR (*P* > 0.05) (Figure 4(b)). Furthermore, the expression of RAB18 was assessed after transfection with miR-455 mimic or inhibitor. The expression of RAB18 was decreased by miR-455 mimic (*P* < 0.05), while it was enhanced by miR-455 inhibitor in Huh7 cells (*P* < 0.05) (Figure 4(c)).

### 3.5. RAB18 Restores Partial Functions of miR-455 on Cell Viability and Invasion

**Figure 5:**
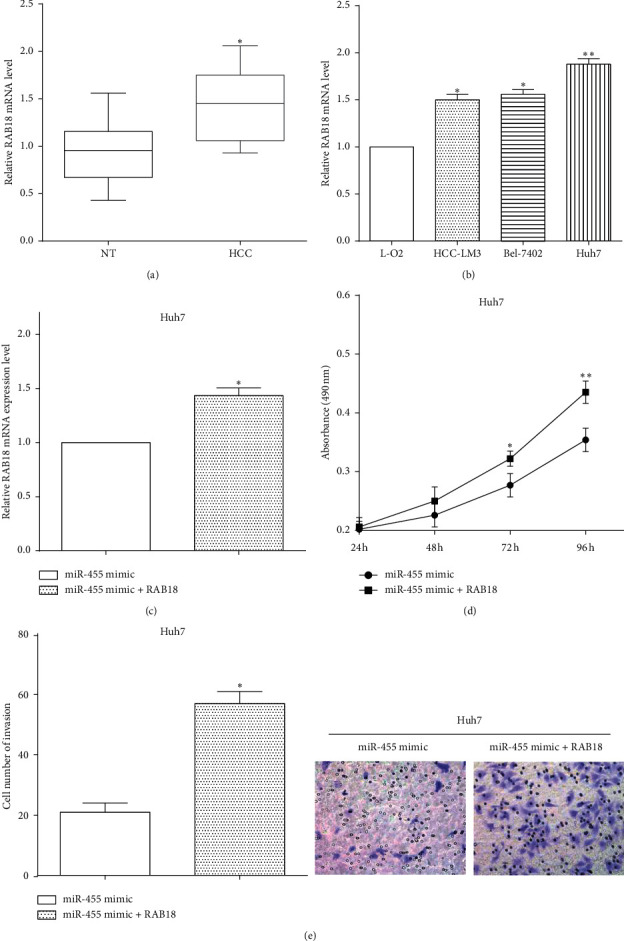
RAB18 restores partial functions of miR-455 on cell viability and invasion. (a) RAB18 was overexpressed in HCC tissues versus nontumor tissues vs NT, ^∗^*P* < 0.05. (b) The expression of RAB18 was higher in HCC-LM3, Huh7, and Bel-7402 than in L-O2 cells vs L-O2, ^∗^*P* < 0.05; ^∗∗^*P* < 0.01. (c) RT-qPCR revealed the transfection efficiency of overexpressing RAB18 in miR-455 mimic-tranfected cells vs miR-455 mimic, ^∗^*P* < 0.05. (d) RAB18 reversed partial functions of miR-455 on cell viability vs miR-455 mimic, ^∗^*P* < 0.05. (e) Overexpressing RAB18 improved cell invasive ability in miR-455 mimic-transfected cells vs miR-455 mimic, ^∗^*P* < 0.05.

To investigate the roles of RAB18 in HCC, the expression of RAB18 was assessed in tissues and cell lines by RT-qPCR. Not unfortunately, RAB18 was overexpressed in HCC tissues versus nontumor tissues (Figure 5(a)). In cells, the expression of RAB18 was higher in HCC-LM3 (*P* < 0.05), Huh7 (*P* < 0.01), and Bel-7402 (*P* < 0.05) cells than in L-O2 cells (Figure 5(b)). To investigate the inhibitory effect of miR-455 on cell proliferation and invasion CCK8 and Transwell assays were performed in miR-455 mimic-transfected Huh7 cells. RAB18 overexpressed plasmid was transfected in miR-455 mimic-transfected cells and the efficiency was calculated by RT-qPCR (Figure 5(c)). CCK8 result revealed that cotransfection with RAB18 overexpressed plasmid and miR-455 mimic increased cell proliferation versus only transfected miR-455 mimic (*P* < 0.05) (Figure 5(d)). Moreover, Transwell assay elucidated that overexpressing RAB18 improved cell invasive ability in miR-455 mimic-transfected cells (*P* < 0.05) (Figure 5(e)). All results validated that RAB18 reversed partial functions of miR-455 on cell viability and invasion in HCC cells.

## 4. Discussion

Although treatment with HCC has improved, recurrence and metastasis often occur, and the 5-year overall survival rate remains low [23, 24]. Therefore, it is important to identify growth and metastasis mechanisms and develop biomarkers that improve patient outcomes.

Increasing evidence indicates that miRNAs have a major impact on cancer growth and metastasis and are associated with tumor development and progression [25, 26]. A previous study showed that miR-455 functioned as a tumor suppressor to inhibit cell proliferation and migration in colorectal cancer [27]. Methods were referred to previous studies [28]. Consistent with the findings in colorectal cancer, we discovered that the expression of miR-455 was low in HCC tissues and cell lines versus the nontumor tissues and normal cells. What is more, our results were consistent with the findings in esophageal squamous cell carcinoma, miR-455 acted as a prognostic marker, and downregulation of miR-455 was connected with worse outcome of HCC patients [29]. miR-455 suppressed cell proliferation of HCC in vitro and in vivo, which was consistent with the findings in breast cancer [30]. What is more, miR-455 impaired cell proliferation and invasion in colorectal cancer [31]. We also found that miR-455 suppressed cell invasive ability in HCC. In addition, miR-455 suppressed the EMT phenomenon of HCC by downregulating N-cadherin expression but upregulating E-cadherin.

RAB18 promoted cell viability, invasion, and migration and impaired cell apoptosis in gastric cancer [32]. In addition, interference of RAB18 suppressed cell viability of non-small-cell lung cancer [33]. In this study, the expression of RAB18 was significantly mediated by miR-455, which was consistent with the findings in gastric cancer where miR-455 targeted RAB18 through directly binding to the 3′-UTR of RAB18 mRNA [34]. What is more, we discovered that RAB18 was overexpressed in HCC tissues and cell lines. RAB18 reversed partial roles of miR-455 on cell viability and invasion in HCC.

## 5. Conclusion

The expression of miR-455 was low in HCC tissues and cell lines, and downregulation of miR-455 was connected with worse outcome of HCC patients. miR-455 suppressed cell growth in vitro and in vivo and suppressed the abilities of cell invasion and EMT in HCC. miR-455 regulated cell viability and invasion by directly targeting the 3′-UTR of RAB18 mRNA. RAB18 reversed partial roles of miR-455 on cell viability and invasion in HCC.

## Figures and Tables

**Table 1:** Primer sequences for RT-qPCR.

Gene	Primer sequences	
*miR-455*	Forward	5′-CGAGCTTCCTTCTGCAGGT-3′
	Reverse	5′-CACCACTGCCATCCCACA-3′

*U6*	Forward	5′-TGCGGGTGCTCGCTTCGCAGC-3′
	Reverse	5′-CCAGTGCAGGGTCCGAGGT-3′

*RAB18*	Forward	5′-CAGGGAAGAAGGCCAAGGAG-3′
	Reverse	5′-CCCGGGGTCGATGGAGT-3′

*GAPDH*	Forward	5′-GAAGGTGAAGGTCGGAGTC-3′
	Reverse	5′-GAAGATGGTGATGGGATTTC-3′

## Data Availability

The data used to support the findings of this study are available from the corresponding author upon request.
